# Supplementary material for: Poor Treatment Outcomes of Locally Advanced Cervical Adenocarcinoma of Human Papilloma Virus Independent Type, Represented by Gastric Type Adenocarcinoma: A Multi-Center Retrospective Study (Sankai Gynecology Study Group)
Source: Cancers (Basel). 2023 Mar 12;15(6):1730. doi: 10.3390/cancers15061730 (PMC10046139; doi:10.3390/cancers15061730)
Supplement: Supplementary file 1 [file cancers-15-01730-s001.zip › cancers-2174929-supplementary.pptx]

## Slide 1
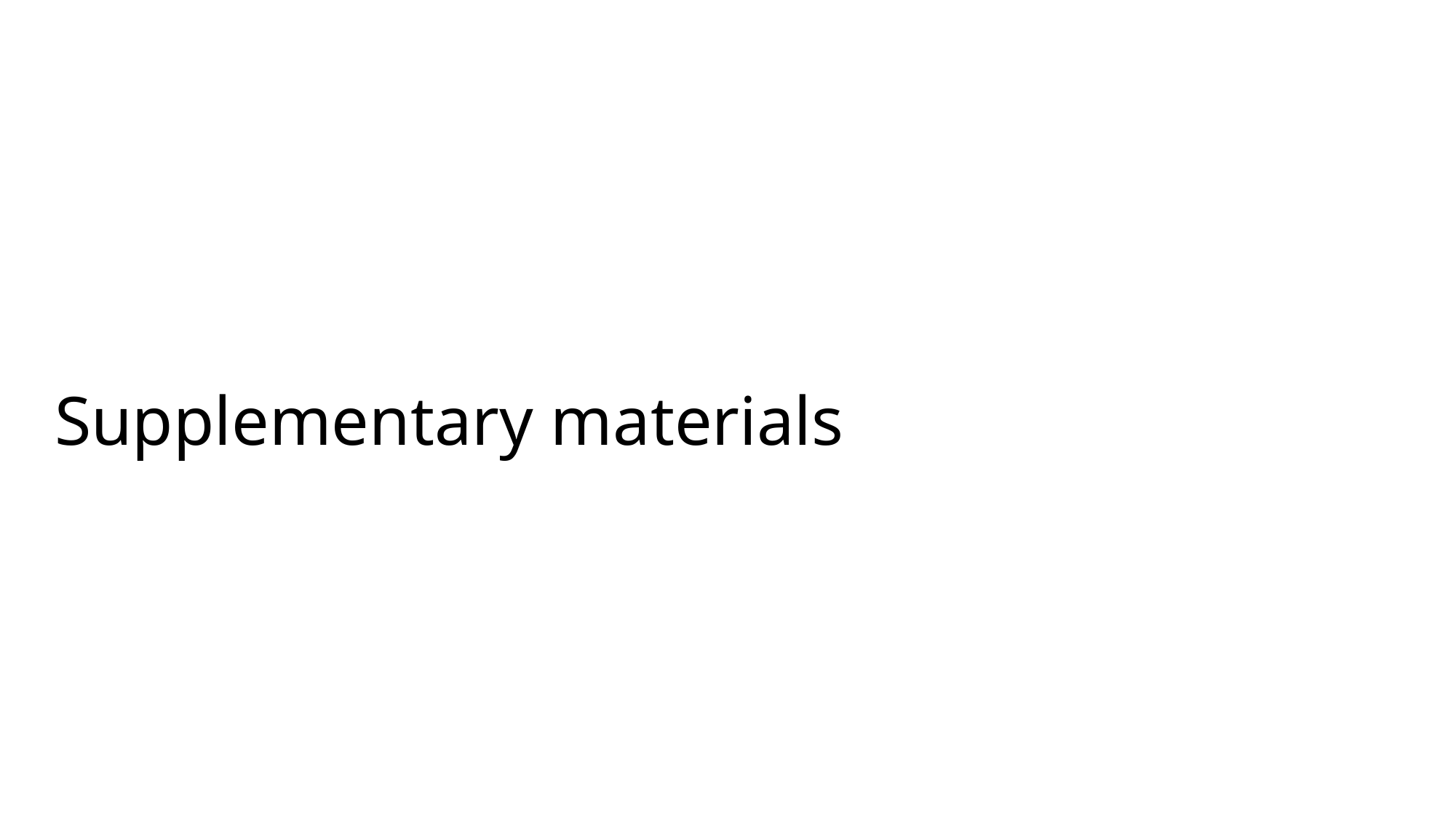

# Supplementary materials

## Slide 2
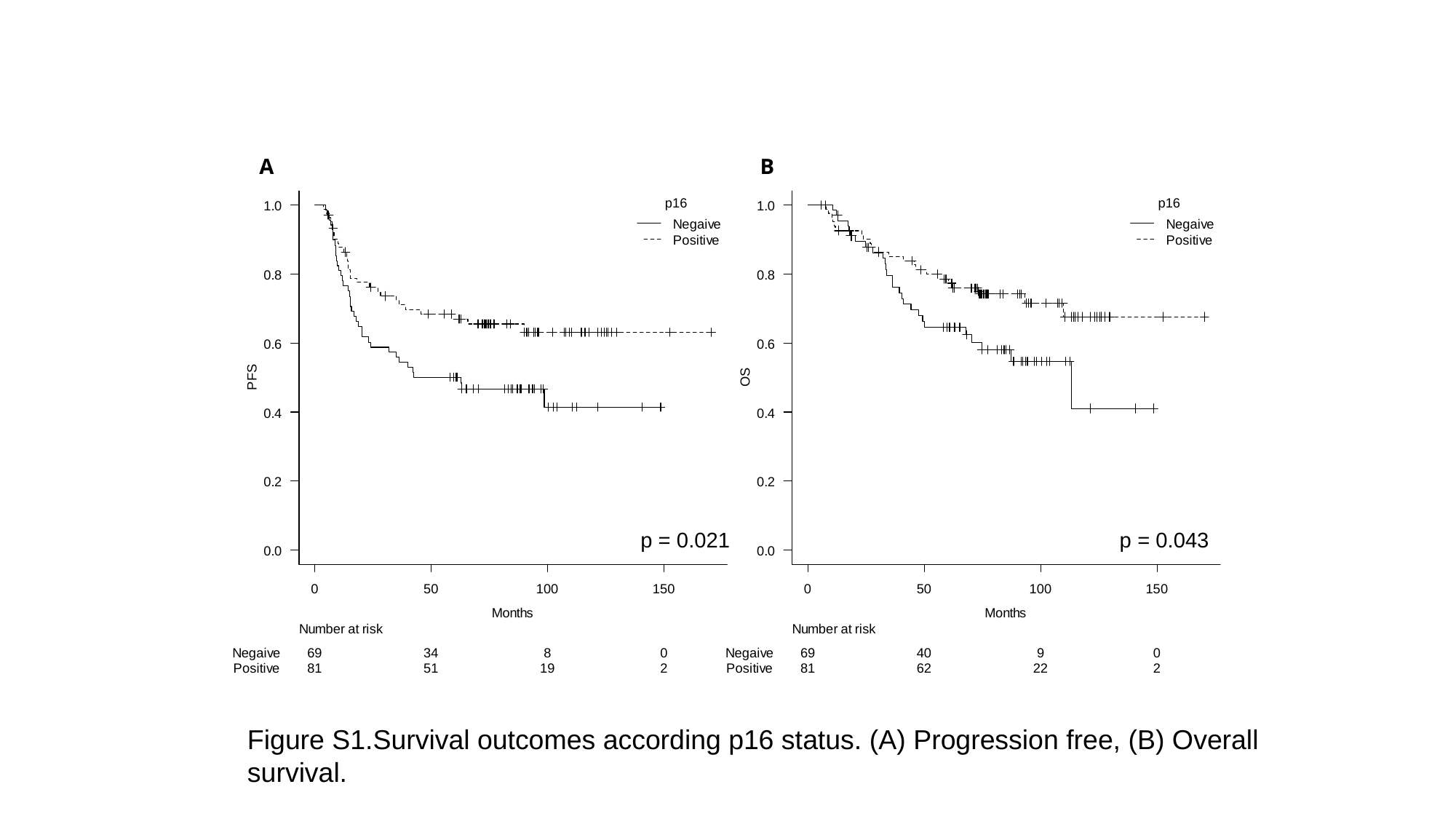

p = 0.021
p = 0.043
A
B
Figure S1.Survival outcomes according p16 status. (A) Progression free, (B) Overall survival.

## Slide 3
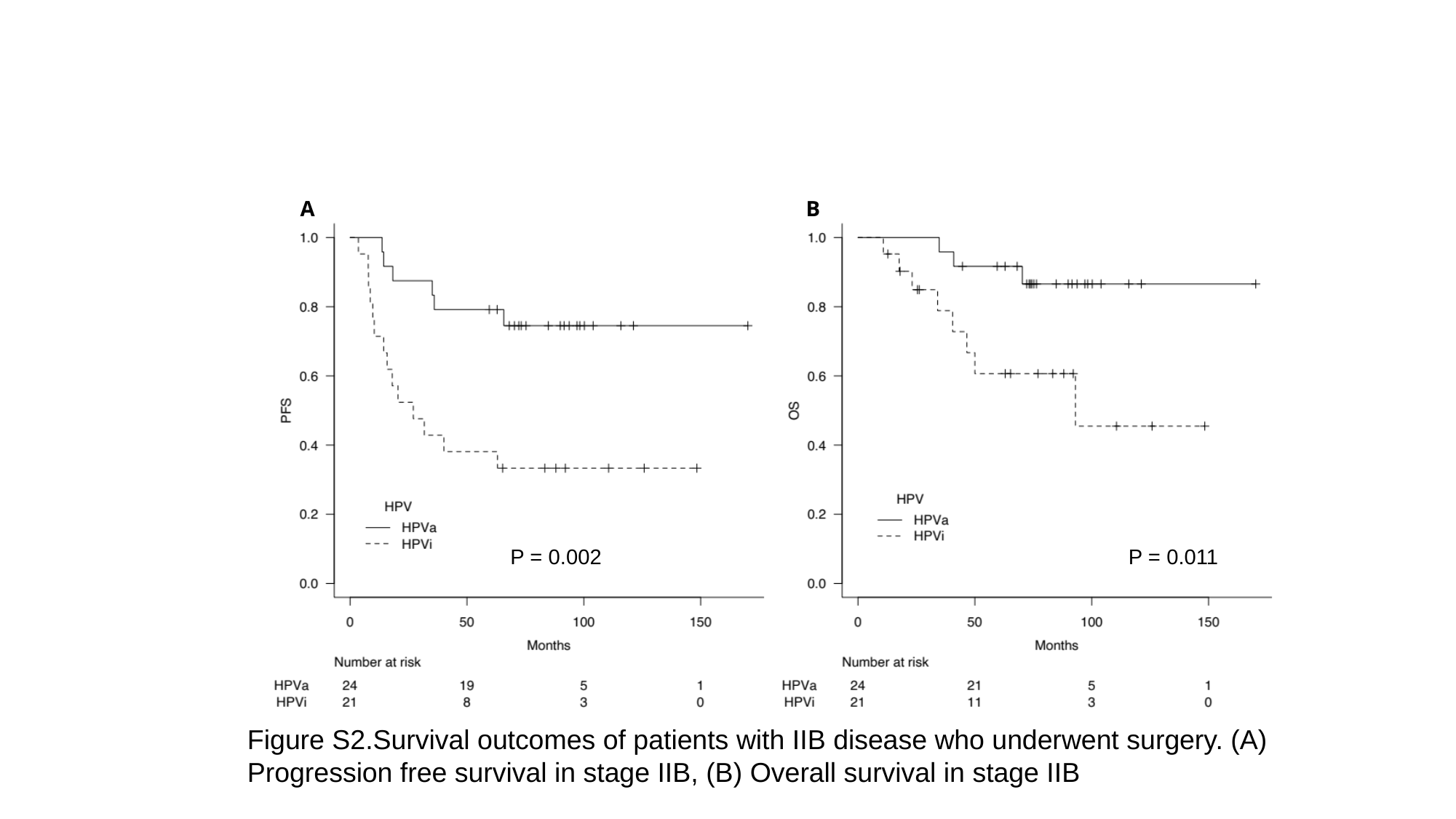

B
A
P = 0.002
P = 0.011
Figure S2.Survival outcomes of patients with IIB disease who underwent surgery. (A) Progression free survival in stage IIB, (B) Overall survival in stage IIB

## Slide 4
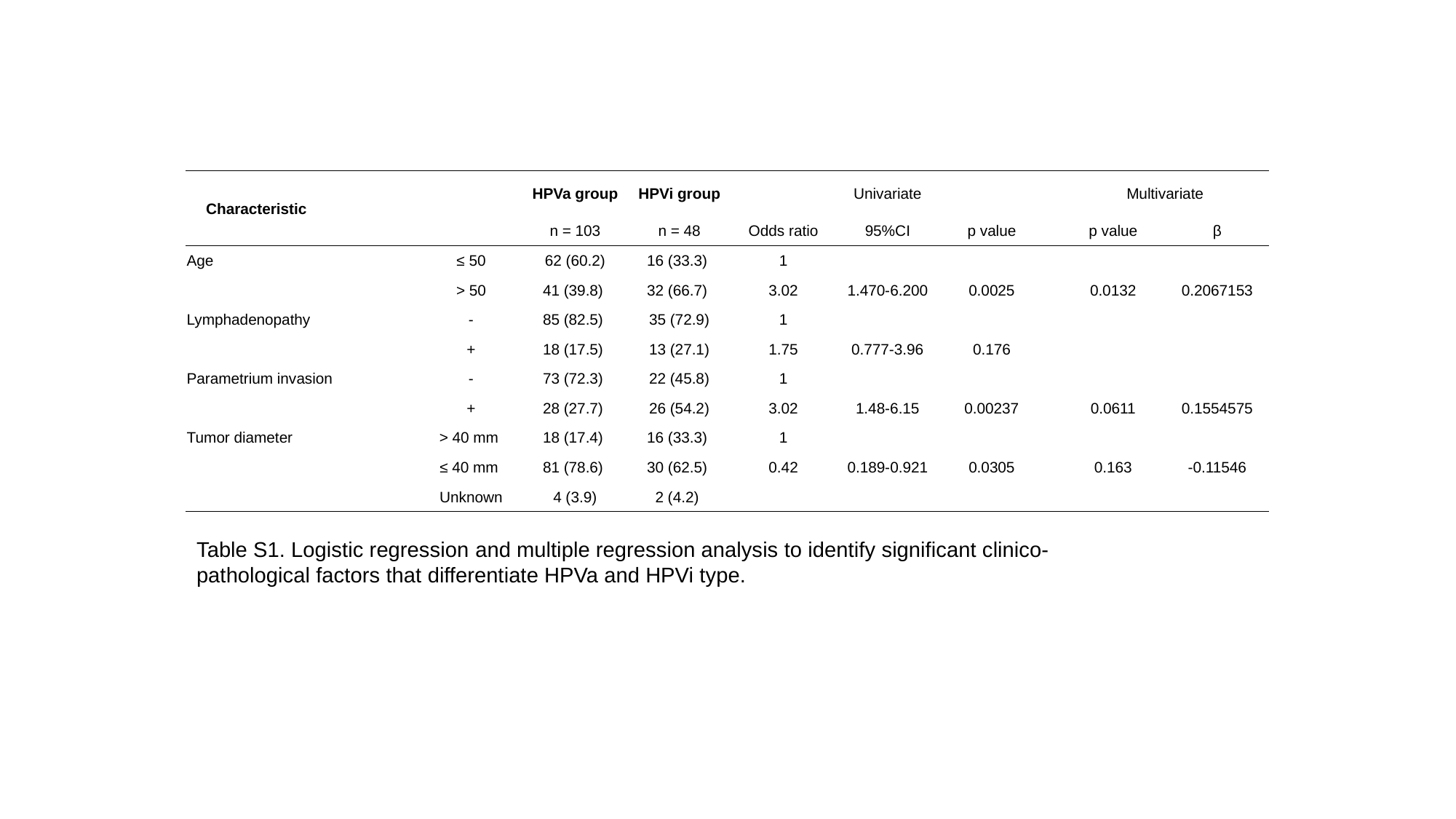

| Characteristic | | HPVa group | HPVi group | Univariate | | | | Multivariate | |
| --- | --- | --- | --- | --- | --- | --- | --- | --- | --- |
| | | n = 103 | n = 48 | Odds ratio | 95%CI | p value | | p value | β |
| Age | ≤ 50 | 62 (60.2) | 16 (33.3) | 1 | | | | | |
| | > 50 | 41 (39.8) | 32 (66.7) | 3.02 | 1.470-6.200 | 0.0025 | | 0.0132 | 0.2067153 |
| Lymphadenopathy | - | 85 (82.5) | 35 (72.9) | 1 | | | | | |
| | + | 18 (17.5) | 13 (27.1) | 1.75 | 0.777-3.96 | 0.176 | | | |
| Parametrium invasion | - | 73 (72.3) | 22 (45.8) | 1 | | | | | |
| | + | 28 (27.7) | 26 (54.2) | 3.02 | 1.48-6.15 | 0.00237 | | 0.0611 | 0.1554575 |
| Tumor diameter | > 40 mm | 18 (17.4) | 16 (33.3) | 1 | | | | | |
| | ≤ 40 mm | 81 (78.6) | 30 (62.5) | 0.42 | 0.189-0.921 | 0.0305 | | 0.163 | -0.11546 |
| | Unknown | 4 (3.9) | 2 (4.2) | | | | | | |
Table S1. Logistic regression and multiple regression analysis to identify significant clinico-pathological factors that differentiate HPVa and HPVi type.

## Slide 5
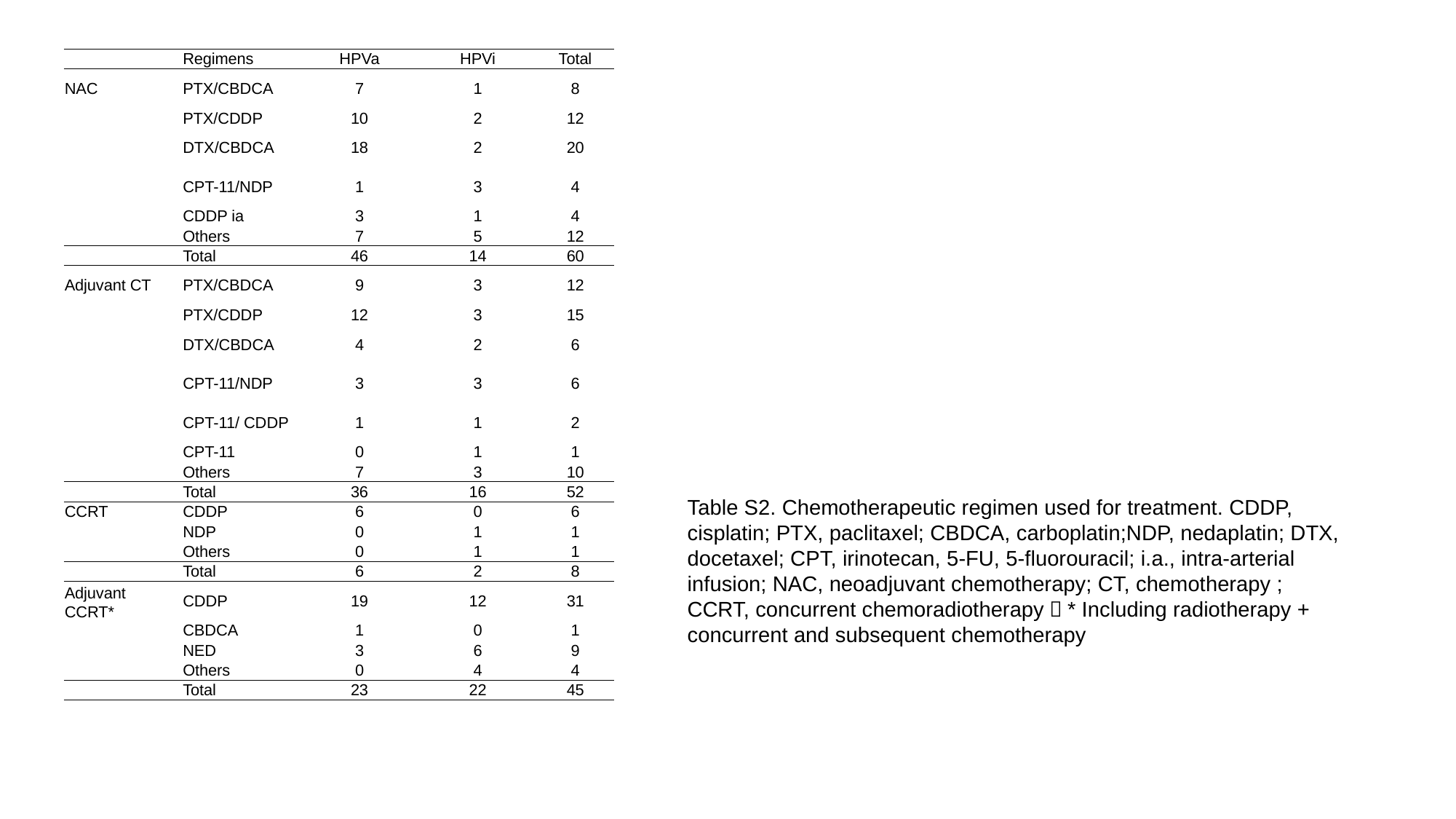

| | Regimens | HPVa | HPVi | Total |
| --- | --- | --- | --- | --- |
| NAC | PTX/CBDCA | 7 | 1 | 8 |
| | PTX/CDDP | 10 | 2 | 12 |
| | DTX/CBDCA | 18 | 2 | 20 |
| | CPT-11/NDP | 1 | 3 | 4 |
| | CDDP ia | 3 | 1 | 4 |
| | Others | 7 | 5 | 12 |
| | Total | 46 | 14 | 60 |
| Adjuvant CT | PTX/CBDCA | 9 | 3 | 12 |
| | PTX/CDDP | 12 | 3 | 15 |
| | DTX/CBDCA | 4 | 2 | 6 |
| | CPT-11/NDP | 3 | 3 | 6 |
| | CPT-11/ CDDP | 1 | 1 | 2 |
| | CPT-11 | 0 | 1 | 1 |
| | Others | 7 | 3 | 10 |
| | Total | 36 | 16 | 52 |
| CCRT | CDDP | 6 | 0 | 6 |
| | NDP | 0 | 1 | 1 |
| | Others | 0 | 1 | 1 |
| | Total | 6 | 2 | 8 |
| Adjuvant　CCRT\* | CDDP | 19 | 12 | 31 |
| | CBDCA | 1 | 0 | 1 |
| | NED | 3 | 6 | 9 |
| | Others | 0 | 4 | 4 |
| | Total | 23 | 22 | 45 |
Table S2. Chemotherapeutic regimen used for treatment. CDDP, cisplatin; PTX, paclitaxel; CBDCA, carboplatin;NDP, nedaplatin; DTX, docetaxel; CPT, irinotecan, 5-FU, 5-fluorouracil; i.a., intra-arterial infusion; NAC, neoadjuvant chemotherapy; CT, chemotherapy ; CCRT, concurrent chemoradiotherapy；* Including radiotherapy + concurrent and subsequent chemotherapy

## Slide 6
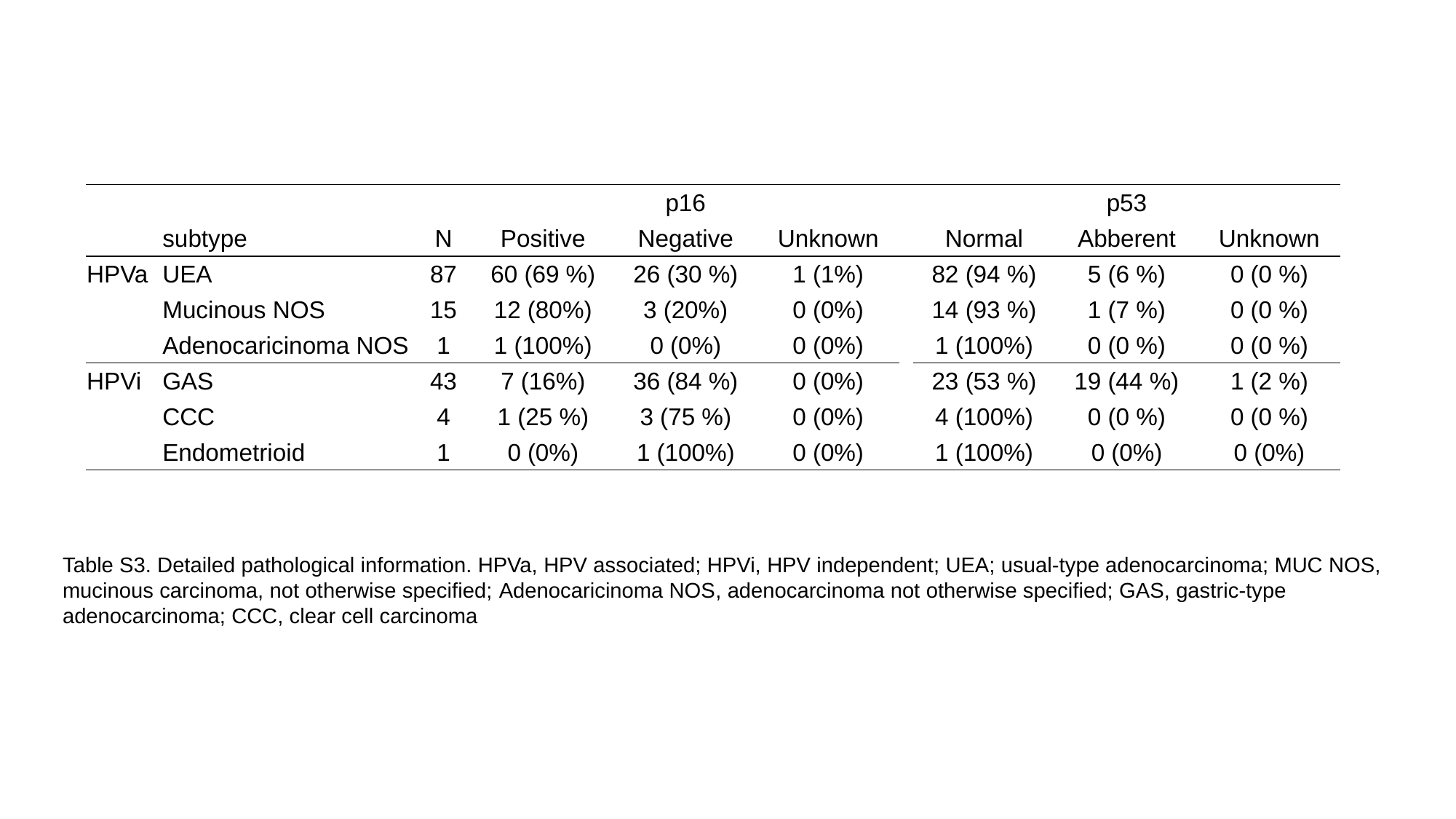

| | | | p16 | | | | p53 | | |
| --- | --- | --- | --- | --- | --- | --- | --- | --- | --- |
| | subtype | N | Positive | Negative | Unknown | | Normal | Abberent | Unknown |
| HPVa | UEA | 87 | 60 (69 %) | 26 (30 %) | 1 (1%) | | 82 (94 %) | 5 (6 %) | 0 (0 %) |
| | Mucinous NOS | 15 | 12 (80%) | 3 (20%) | 0 (0%) | | 14 (93 %) | 1 (7 %) | 0 (0 %) |
| | Adenocaricinoma NOS | 1 | 1 (100%) | 0 (0%) | 0 (0%) | | 1 (100%) | 0 (0 %) | 0 (0 %) |
| HPVi | GAS | 43 | 7 (16%) | 36 (84 %) | 0 (0%) | | 23 (53 %) | 19 (44 %) | 1 (2 %) |
| | CCC | 4 | 1 (25 %) | 3 (75 %) | 0 (0%) | | 4 (100%) | 0 (0 %) | 0 (0 %) |
| | Endometrioid | 1 | 0 (0%) | 1 (100%) | 0 (0%) | | 1 (100%) | 0 (0%) | 0 (0%) |
Table S3. Detailed pathological information. HPVa, HPV associated; HPVi, HPV independent; UEA; usual-type adenocarcinoma; MUC NOS, mucinous carcinoma, not otherwise specified; Adenocaricinoma NOS, adenocarcinoma not otherwise specified; GAS, gastric-type adenocarcinoma; CCC, clear cell carcinoma

## Slide 7
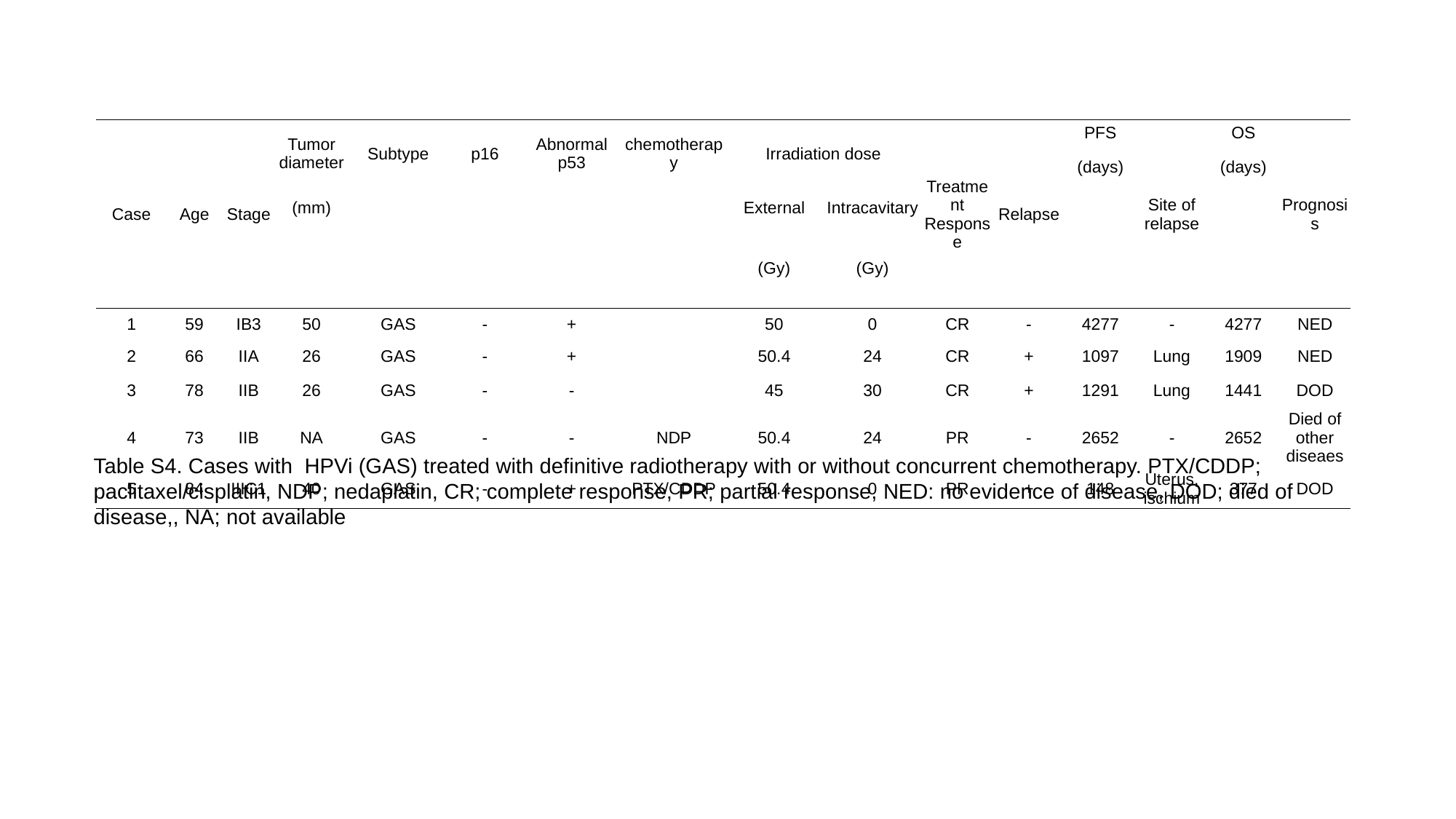

| Case | Age | Stage | Tumor diameter | Subtype | p16 | Abnormal p53 | chemotherapy | Irradiation dose | | Treatment Response | Relapse | PFS | Site of relapse | OS | Prognosis |
| --- | --- | --- | --- | --- | --- | --- | --- | --- | --- | --- | --- | --- | --- | --- | --- |
| | | | | | | | | | | | | (days) | | (days) | |
| | | | (mm) | | | | | External | Intracavitary | | | | | | |
| | | | | | | | | (Gy) | (Gy) | | | | | | |
| 1 | 59 | IB3 | 50 | GAS | - | + | | 50 | 0 | CR | - | 4277 | - | 4277 | NED |
| 2 | 66 | IIA | 26 | GAS | - | + | | 50.4 | 24 | CR | + | 1097 | Lung | 1909 | NED |
| 3 | 78 | IIB | 26 | GAS | - | - | | 45 | 30 | CR | + | 1291 | Lung | 1441 | DOD |
| 4 | 73 | IIB | NA | GAS | - | - | NDP | 50.4 | 24 | PR | - | 2652 | - | 2652 | Died of other diseaes |
| 5 | 84 | IIIC1 | 40 | GAS | - | + | PTX/CDDP | 50.4 | 0 | PR | + | 148 | Uterus, ischium | 377 | DOD |
Table S4. Cases with HPVi (GAS) treated with definitive radiotherapy with or without concurrent chemotherapy. PTX/CDDP; paclitaxel/cisplatin, NDP; nedaplatin, CR; complete response, PR; partial response, NED: no evidence of disease, DOD; died of disease,, NA; not available
